# A Quick Sequential Organ Failure Assessment (qSOFA) Score Greater than 1 and Shortened Ampicillin Use Predict Death and One-Year Mortality in Hospitalized Patients with Non-Perinatal Invasive Listeriosis: A Retrospective Analysis of 118 Consecutive Cases

**DOI:** 10.3390/microorganisms12112365

**Published:** 2024-11-19

**Authors:** Shuh-Kuan Liau, Cheng-Chieh Hung, Chao-Yu Chen, Yi-Chun Liu, Yueh-An Lu, Yu-Jr Lin, Yung-Chang Chen, Ya-Chung Tian, Fan-Gang Tseng, Hsiang-Hao Hsu

**Affiliations:** 1 Department of Nephrology, Kidney Research Center, Chang Gung Memorial Hospital, Linkou Branch, College of Medicine, Chang Gung University, Taoyuan 333, Taiwan; stevenliau@cgmh.org.tw (S.-K.L.); b9102033@cgmh.org.tw (C.-Y.C.); yiichun@cgmh.org.tw (Y.-C.L.); trueth@cgmh.org.tw (Y.-A.L.); cyc2356@cgmh.org.tw (Y.-C.C.); dryctian@cgmh.org.tw (Y.-C.T.); 2 Research Services Center for Health Information, Chang Gung University, Taoyuan 33302, Taiwan; doublelin15@gmail.com; 3 Department of Engineering and System Science, Frontier Research Center on Fundamental and Applied Sciences of Matters, National Tsing Hua University, Hsinchu 30013, Taiwan; fangang@ess.nthu.edu.tw; 4 Institute of Nano Engineering and Microsystems, National Tsing Hua University, Hsinchu 30013, Taiwan

**Keywords:** listeriosis, mortality, prognosis, qSOFA, risk factors

## Abstract

*Listeria monocytogenes* causes listeriosis, a serious foodborne illness with a high mortality rate, especially in vulnerable populations. It accounts for 19% of foodborne deaths, with invasive cases having a mortality rate of up to 44%, leading to conditions like meningitis, bacteremia, and meningoencephalitis. However, the prognostic factors remain unclear. This study examines the hospital outcomes of invasive listeriosis and identifies risk factors for in-hospital and one-year mortality. We analyzed the electronic medical records of 118 hospitalized patients with non-perinatal, culture-proven invasive listeriosis collected over a 21-year period. The in-hospital mortality rate was 36.4%, with only 33.1% surviving one year and 22.0% surviving two years. The key findings indicate that a quick Sequential Organ Failure Assessment (qSOFA) score of ≥2 (OR 106.59, *p* < 0.001), respiratory failure (OR 7.58, *p* = 0.031), and shorter ampicillin duration (OR 0.53, *p* = 0.012) independently predicted poorer in-hospital outcomes. Additionally, a qSOFA score of ≥2 (OR 8.46, *p* < 0.001) and shorter ampicillin duration (OR 0.65, *p* < 0.001) were linked to higher one-year mortality. This study is the first to identify a qSOFA score of ≥2 as a significant marker for high-risk invasive listeriosis patients, with poorer outcomes linked to a qSOFA score of ≥2, respiratory failure, and shorter ampicillin use.

## 1. Introduction

The causative agent of listeriosis, *Listeria monocytogenes*, is a facultative anaerobic bacterium. The main source of infection for *Listeria* is through eating food or drinking water contaminated with its bacteria. *Listeria* contamination is a major concern associated with foods such as unwashed raw fruits and vegetables, soft cheeses made from raw (unpasteurized) milk, hot dogs, luncheon meats, cold cuts, fermented or dry sausage, and store-made chilled salads [1,2].

Listeriosis is a foodborne illness with severe public health implications. However, it is relatively uncommon and avoidable despite its high fatality rate if not properly treated. In a study of foodborne infections in the United States, *Listeria monocytogenes* had the third highest mortality rate at 16%, just below those of *Vibrio vulnificus* (35%) and *Clostridium botulinum* (17%), and it was responsible for almost 19% of all foodborne infection-related deaths [3]. In an analysis conducted in Britain, the death rate of listeriosis unrelated to pregnancy was even higher, reaching up to 44% [4].

When it spreads outside of the intestines, *Listeria* can cause invasive listeriosis, which includes bacteremia, septicemia, meningitis, and meningoencephalitis [5]. On the other hand, most systemic infections strike hosts who are immunocompromised, old, neonatal, or pregnant—those with impaired or immature immune systems [6]. Anemia, diabetes mellitus, kidney or liver disease, hemochromatosis, taking antacids or antibiotics, being older, having cancer, using immunosuppressive medications (such as steroids), drinking excessively, and having other conditions that impair immunity in the intestines or throughout the body are all at higher risk of developing invasive listeriosis according to a literature review [7,8]. Nonetheless, reports on prognostic factors are scarce in this population.

In a nationwide prospective observational cohort study involving 818 French cases, the most significant death predictors were persistent malignancy, multi-organ failure, the worsening of any pre-existing organ dysfunction, and monocytopenia for both bacteremia and neurolisteriosis [9]. In a study of 281 cases of non-perinatal listeriosis, Guevara et al. found statistically significant risk factors for death. These included non-hematological cancer, heavy drinking, being over 70 years old, using steroids, and having kidney disease [7]. Despite reports from Europe and the United States, the prognostic factors for invasive listeriosis in East Asian countries, such as Taiwan, are poorly defined. It has also not been determined how the quick Sequential Organ Failure Assessment (qSOFA) score can be used in invasive listeriosis. The qSOFA score is a simple number comprising a respiratory rate (RR) greater than or equal to 22 breaths per minute, altered mentation (GCS < 15), and systolic blood pressure (SBP) less than 100 mmHg. It is a good predictor of survival in people with sepsis and septic shock.

This retrospective study aimed to identify the risk factors for non-perinatal invasive listeriosis fatalities to improve patients’ dismal prognosis and initiate proper treatment sooner. We also attempted to evaluate the predictive validity of the qSOFA score in determining the risk of death in this population.

## 2. Materials and Methods

### 2.1. Study Design and Patient Selection

We retrospectively examined the medical records of patients admitted to Linkou Chang Gung Memorial Hospital, a tertiary medical center, for invasive listeriosis between 1 August 2000 and 31 August 2021. With approximately 3700 beds, Linkou Chang Gung Memorial Hospital serves about 4 million outpatient visits, 200,000 emergency visits, and 100,000 inpatient admissions annually. We used ICD-9-CM code 0270 for discharge diagnosis to check for invasive listeriosis. Other ICD-10-CM codes (A32, A320, A3211, A3212, A327, A328, and A339) were used to check for listeriosis, cutaneous listeriosis, listerial meningitis, listerial meningoencephalitis, listerial sepsis, other types of listeriosis, and unspecified listeriosis. All patients included in this study had invasive listeriosis. Invasive listeriosis is defined as a *Listeria* infection that has spread beyond the intestines to normally sterile sites, resulting in conditions including bacteremia, meningitis, and meningoencephalitis. A positive culture result for *Listeria monocytogenes* in blood or cerebrospinal fluid (CSF) allowed us to confirm the diagnosis of invasive listeriosis. We excluded six perinatal cases from the analysis because their age was less than seven days after birth. This study identified and enrolled 118 patients with invasive listeriosis over a 21-year period.

The Chang Gung Medical Foundation’s Institutional Review Board approved the study (approval number: 202300976B0) and waived the requirement for each participant’s written informed consent because the study anonymized their personal information.

### 2.2. Patient Characteristics and Outcomes

We obtained information on age, sex, associated comorbidities (diabetes mellitus, hypertension, coronary artery disease, heart failure, atrial fibrillation, cirrhosis, peptic ulcer disease, cerebral vascular accident, chronic obstructive airway disease, solid cancer, hematologic malignancy, autoimmune diseases, organ transplant status, and chronic dialysis), immunosuppressant use, and anti-cancer treatment. We recorded fever, nausea or vomiting, myalgia, abdominal pain, diarrhea, headache, confusion, and dyspnea as symptoms and signs of invasive listeriosis in the clinical information. The qSOFA score was collected as a simple score comprising three elements: RR ≥ 22 breaths per minute, altered mentation (GCS < 15), and SBP ≤ 100 mmHg [10]. We documented the results of blood tests performed upon admission and on the seventh day of hospitalization. All patients had blood cultures taken, and we documented the length of time they received ampicillin and other potent antibiotics, like penicillin G, piperacillin–tazobactam, amoxicillin–clavulanate, gentamicin, meropenem, trimethoprim–sulfamethoxazole, and linezolid [11]. We quantified the treatment delay for listeriosis as the interval between the confirmation of diagnosis and the initiation of ampicillin therapy, the primary treatment regimen for invasive listeriosis [12,13]. We examined each patient’s medical records for at least two years, from the time of admission to confirmed death.

We used the following approach to ascertain the one-year mortality rate, another important outcome: One year after discharge, if there were still blood tests, examinations, or records of dialysis therapy, outpatient care, hospitalization, etc., we determined that the patient had survived for more than a year. We contacted the patient to confirm their survival if their electronic medical records did not record their death within a year of their discharge.

### 2.3. Statistical Analysis

A retrospective cohort study was used for this investigation. The presentation of demographic and clinical data includes means and standard deviations (SD) for continuous data, as well as counts and percentages (%) for categorical data. To compare continuous and categorical factors between survivors and non-survivors, a T-test was used for continuous variables, while categorical data were analyzed using the Chi-square test. If sample sizes were too small, Fisher’s exact test was applied instead.

We used the logistic regression and Cox proportional hazard models in the multivariate analyses to identify the risk factors for in-hospital and one-year mortality. Calculations were conducted on the variables determined to be significant in the univariate analysis. We plotted Kaplan–Meier survival curves for groups with qSOFA scores of less than 2 or 2 or greater.

We utilized the statistical software R 3.0.2 (Copyright, the R Foundation for Statistical Computing, Vienna, Austria). All *p*-values reported were two-sided, and *p* < 0.05 was deemed to indicate statistical significance.

## 3. Results

### 3.1. Demographic Information and Outcomes of Hospitalized Patients with Invasive Listeriosis

This investigation included 118 hospitalized individuals with culture-confirmed invasive listeriosis. Table 1 displays a male-to-female ratio of 1:1.15 and an average age of 60.0 ± 18.4 years. There were no differences between survivors and non-survivors in terms of their age or gender distribution. In total, 75 patients (63.6%) survived hospitalization, while 43 patients (36.4%) did not. A year after hospitalization, only 39 patients (33.1%) remained alive. Only 26 individuals (22.0%) were still alive two years after admission. Those who left the hospital alive had an average age of 58.7 ± 18.5 years, while those who did not had an average age of 62.4 ± 18.3 years (*p* = 0.294).

Out of 118 hospitalized patients with invasive listeriosis, 47.5% had high blood pressure, 36.4% had peptic ulcer disease, 33.1% had solid cancer, 26.3% had diabetes, 24.6% had an autoimmune disease, 22% were on long-term dialysis, 19.5% had cirrhosis, 16.9% had hematologic cancer, 16.1% had heart failure, 12.7% had atrial fibrillation, 9.3% had a cerebrovascular accident, 8.5% had chronic obstructive airway disease, 5.9% had coronary artery disease, and 5.1% had an organ transplant. The most prevalent comorbidities were hypertension and peptic ulcer disease. Aside from peptic ulcer disease, there were no major differences in baseline comorbidities between people who survived and people who did not survive. However, there was a large difference in the number of people who had peptic ulcer disease between people who survived (26.7%, n = 20) and people who did not survive (53.5%, n = 23; OR 3.16, 95% CI 1.45–7.05, *p* = 0.004). In terms of concurrent therapy at baseline, 56.8% of patients received immunosuppressants, 12.7% received chemotherapy in the four weeks prior, and 10.2% received radiotherapy in the four weeks prior. Overall, there was no significant difference between survivors and non-survivors in comorbid therapy at baseline.

### 3.2. Clinical Characteristics of Hospitalized Patients with Invasive Listeriosis

Table 2 details the symptoms and signs observed, including 94.1% fever, 67.8% nausea/vomiting, 55.9% dyspnea, 44.9% confusion, 44.1% myalgia, 39% abdominal pain, 33.1% diarrhea, and 31.4% headache. While confusion (18.7% vs. 90.7%, OR 42.48, 95% CI 14.38–160.0; *p* = 0.000) and dyspnea (46.7% vs. 72.1%, OR 2.95, 95% CI 1.34–6.80; *p* = 0.008) were substantially more common among non-survivors, nausea/vomiting (76% vs. 53.5%, OR 0.36, 95% CI 0.16–0.80; *p* = 0.013), myalgia (53.3% vs. 27.9%, OR 0.34, 95% CI 0.15–0.74; *p* = 0.008), and headache (41.3% vs. 14.0%, OR 0.23, 95% CI 0.08–0.58; *p* = 0.003) were much more frequent among survivors.

In terms of complications, shock (12% vs. 67.4%, OR 15.19, 95% CI 6.14–41.08; *p* = 0.000) and respiratory failure (17.3% vs. 86.1%, OR 29.41, 95% CI 10.97–91.50; *p* = 0.000) differed significantly between the two groups. We collected the components of the qSOFA score, which has previously served as a predictor of survival. A higher risk of death in the hospital was found in people with a blood pressure of ≤100 mmHg (21.3% vs. 81.4%, OR 16.13, 95% CI 6.55–44.05; *p* = 0.000), altered mental status (GCS < 15) (18.7% vs. 90.7%, OR 42.48%, 95% CI 14.38–160.00; *p* = 0.000), a respiratory rate of ≥22 breaths per minute (21.3% vs. 93%, OR 49.17, 95% CI 15.39–222.52; *p* = 0.000), and a qSOFA score ≥ 2 (7.0% vs. 93.3%, OR 186.67, 95% CI 48.96–1005.23; *p* = 0.000).

We recorded each patient’s hemogram and biochemical data on days 1 and 7 of hospitalization. On the first day of admission, blood urea nitrogen (BUN) levels (35.84 ± 36.39 mg/dL vs. 56.71 ± 55.25 mg/dL, OR 1.01, 95% CI 1.00–1.02, *p* = 0.032) and C-reactive protein (CRP) (71.19 ± 70.60 mg/L vs. 125.56 ± 93.61 mg/L, OR 1.01, 95% CI 1.00–1.01; *p* = 0.002) were much higher in non-survivors than in survivors. Serum albumin levels were also much lower in survivors than in non-survivors (3.05 ± 0.67 g/dL vs. 2.59 ± 0.60 g/dL, OR 0.31, 95% CI 0.13–0.66; *p* = 0.004). On day 7 of hospitalization, a higher polymorphonuclear (PMN) leukocyte percentage (71.64 ± 21.41% vs. 82.60 ± 13.55%, OR 1.04, 95% CI 1.01–1.98; *p* = 0.008), an absolute neutrophil count (ANC) (5820.09 ± 3649.37/μL vs. 9511.50 ± 6236.94/μL, OR 1.00, 95% CI 1.00–1.00; *p* = 0.001), higher BUN (29.41 ± 24.17 mg/dL vs. 53.62 ± 45.73 mg/dL, OR 1.01, CI 1.01–1.04; *p* = 0.004), higher potassium (3.80 ± 0.59 mEq/L vs. 4.15 ± 0.87, OR 2.00, 95% CI 1.14–3.76; *p* = 0.021), higher CRP (56.38 ± 64.23 mg/dL vs. 94.62 ± 85.95 mg/dL, OR 1.01, 95% CI 1.00–1.01; *p* = 0.020), lower albumin (2.84 ± 0.50 g/dL vs. 2.38 ± 0.35 g/dL, OR 0.07, 95% CI 0.01–0.31; *p* = 0.002), and higher total bilirubin (0.93 ± 0.80 mg/dL vs. 3.11 ± 4.93, OR 1.44, 95% CI 1.11–2.18; *p* = 0.033) were associated with an increased risk of in-hospital mortality.

The length of ampicillin therapy was recorded. A shorter ampicillin administration duration was significantly associated with increased in-hospital mortality (2.2 ± 1.16 weeks vs. 1.6 ± 1.5 weeks, OR 0.76, 95% CI 0.58–0.98; *p* = 0.044).

Additionally, we found no significant difference between survivors and non-survivors when we incorporated all effective antibiotics into the analysis and assessed the treatment durations. In total, 22% of our study population received gentamicin synergy therapy, and its use did not show a statistically significant difference in mortality between survivors and non-survivors. The treatment delay for ampicillin therapy was significantly longer in non-survivors than survivors (1.0 ± 1.9 days vs. 2.1 ± 2.5 days, OR 1.26, 95% CI 1.06–1.54; *p* = 0.015).

All 118 patients had invasive listeriosis that was proven to be caused by a pathogen. Of these, 117 (97.5%) had *Listeria* bacteremia, and 11 (9.3%) had an infection in the central nervous system (CNS). Combined bacterial infections occurred with 7.6% CoNS, 5.1% *E. coli*, 4.2% candidemia, 1.7% *Klebsiella pneumoniae*, 0.8% *Enterobacter cloacae*, 0.8% *Pseudomonas aeruginosa*, and 0.8% Methicillin-resistant *Staphylococcus aureus* (MRSA) bacteremia. Only those who also had *E. coli* bacteremia experienced significantly higher mortality than those who did not (1.3% vs. 11.6%, OR 9.74, 95% CI 1.50–190.12; *p* = 0.041).

### 3.3. Multivariate Analyses of Risk Factors for In-Hospital and One-Year Mortality in Hospitalized Patients with Invasive Listeriosis

**Figure 1:**
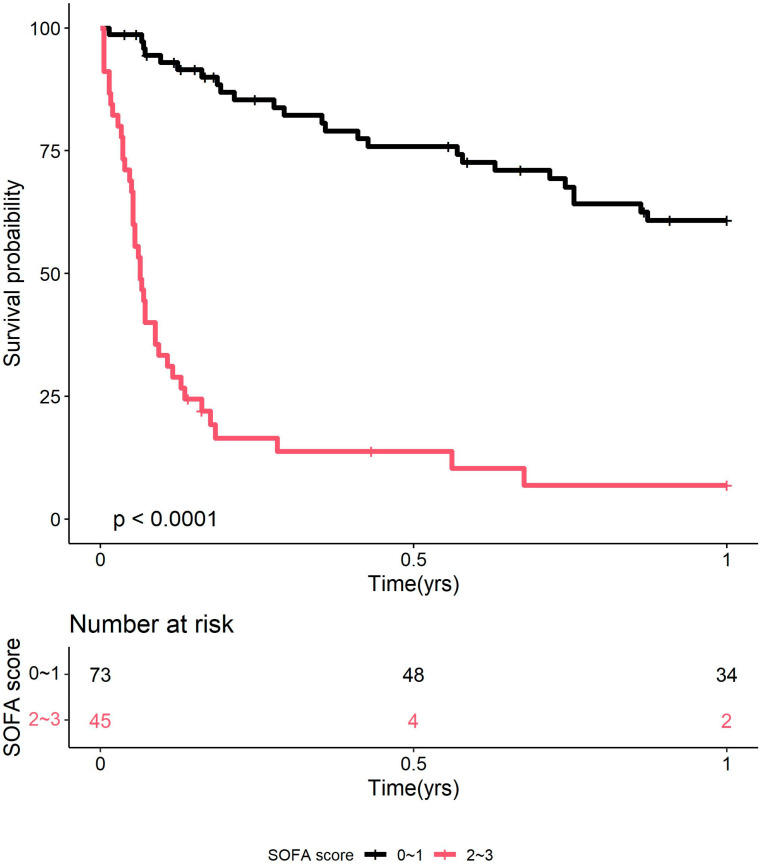
Kaplan–Meier plot showing in-hospital survival of patients with invasive listeriosis, stratified by qSOFA score (0–1 vs. ≥2). **Abbreviations:** qSOFA, quick Sequential Organ Failure Assessment.

We identified significant in-hospital mortality factors using multivariate logistic regression and Cox’s proportional hazards model (Table 3). Our study found that having a qSOFA score of 2 or more (OR 106.59, 95% CI 17.26–1136.26; *p* = 0.000), respiratory failure (OR 7.58, 95% CI 1.14–52.69; *p* = 0.031), and using ampicillin for less than two weeks (OR 0.53, 95% CI 0.31–0.84; *p* = 0.012) were all independently linked to death in the hospital. A qSOFA score ≥ 2 (OR 8.46, 95% CI 3.84–18.64; *p* = 0.000) and reduced duration of ampicillin use (OR 0.65, 95% CI 0.54–0.78; *p* = 0.000) were independently associated with worse in-hospital outcomes for one-year mortality. The Kaplan–Meier analysis (Figure 1) reveals that patients with a qSOFA score of ≥2 had a substantially worse in-hospital outcome than those with a score of <2 (*p* < 0.0001).

## 4. Discussion

Given the paucity of prior studies, we retrospectively evaluated differences in in-hospital and one-year mortality between survivors and non-survivors among patients with invasive listeriosis. The current investigation identified several prognostic variables. As determined using univariate analysis, the patients’ outcomes in the hospital were worse when they had peptic ulcer disease; confusion; dyspnea; shock; respiratory failure; SBP ≤ 100 mmHg; an altered mental state according to the Glasgow Coma Scale; an RR ≥ 22; a qSOFA score of ≥2; higher BUN/CRP and a lower albumin level on day 1 of hospitalization; a higher PMN percentage/ANC/potassium/CRP/bilirubin and lower albumin level one week after admission; a shorter ampicillin use duration; a longer treatment delay for ampicillin therapy; and combined *E. coli* bacteremia.

Unexpectedly, hospitalized patients with nausea/vomiting, myalgia, and headaches survived better than those without these symptoms. Except for peptic ulcer disease, there were no differences in age, gender, or baseline comorbidities between survivors and non-survivors. After multivariate analysis, a qSOFA score of ≥2, respiratory failure, and a shorter ampicillin usage duration were associated with higher in-hospital mortality; a qSOFA score of ≥2 and shorter ampicillin usage duration were also associated with higher one-year mortality. Our findings emphasize the necessity for early and adequate care once patients with invasive listeriosis have progressed beyond the manifestations mentioned above.

Our 36.4% in-hospital mortality rate is consistent with the 16–44% range reported in the literature [3,4,7,14,15,16]. Previous studies show that patients with invasive listeriosis older than 70 have a significantly higher risk of dying [7], but our study did not observe any age differences between survivors and non-survivors. The average age of our cohort was 60.0 ± 18.4, possibly explaining the less pronounced influence of age on mortality.

Among the 75 patients who initially survived hospitalization, 36 (48.0%) passed away within the first year of follow-up, and this number increased to 49 (65.3%) within the two-year follow-up period. The causes of death among these 49 patients were mostly infection or sepsis (69.4%), followed by cancer (16.3%), autoimmune diseases (8.2%), and cardiovascular diseases (4.1%), and one patient (2.0%) died from unknown causes. These findings show that while patients may survive the acute infection, their health remains severely compromised, leading to a shortened life expectancy.

Previous studies have linked proton pump inhibitors (PPIs) to an increased risk of listeriosis. Gillespie et al. analyzed 780 cases of non-pregnancy-associated listeriosis in England and Wales, finding that the rise in prescriptions for PPIs from 1998 to 2007 closely matched an increase in *L. monocytogenes* bacteremia [17]. Later, Jensen et al. studied 721 adult patients with listeriosis and found that the adjusted odds ratio for current PPI use and listeriosis development was 2.81, with PPI use up to 90 days before diagnosis still being significant [18]. The increased gastric pH from PPI use is thought to weaken the body’s defense against *Listeria monocytogenes*. Animal studies by Schlech III et al. showed that reduced gastric acidity can lower the infection dose needed for invasive listeriosis [19]. In our study, non-survivors had a higher prevalence of peptic ulcer disease than survivors. We assume that patients with peptic ulcer disease may use more gastric-acid-lowering agents, which may impair the host’s natural defense against *Listeria monocytogenes*, leading to higher mortality. There is still a need to conduct further research on the connection between listeriosis, peptic ulcer disease, and acid-lowering medications. We now recommend aggressive antibiotic therapy for patients with peptic ulcer disease who develop invasive listeriosis, as we anticipate a poorer clinical outcome.

Shi et al. found that the most common symptoms of listeriosis were fever (91.7%) and altered consciousness (50.0%), followed by rashes (45.8%), respiratory distress (37.5%), nuchal rigidity (29.2%), and headaches (20.8%) [20]. In our study, fever was also the most common symptom (111 of 118 or 94.1%), but there was no significant difference in fever rates between survivors and non-survivors. Additionally, we also discovered that myalgia (44.1%), headache (31.4%), and nausea/vomiting (67.8%) were common symptoms. Interestingly, patients with these symptoms tended to have better in-hospital outcomes. Patients with milder symptoms may have more serious underlying conditions or weakened immune systems, which can lead to more severe symptoms, like confusion, difficulty breathing, and shock, possibly explaining these unexpected results.

In 2016, the third international task force changed the definition of sepsis and suggested using the qSOFA score (range, 0–3 points, with 1 point for systolic hypotension [≤100 mm Hg], tachypnea [≥22/min] or altered mentation [GCS < 15]) instead of the SIRS criteria to identify patients at high risk of death [21]. Studies show that qSOFA is better at predicting hospital death than SOFA or SIRS. A qSOFA score of 2 or higher is associated with a 3- to 14-fold increased risk of death in hospital [10,22]. However, the predictive value of qSOFA for invasive listeriosis patients remains unclear. In our study, we found that patients with a qSOFA score of ≥2 had 103 times higher in-hospital mortality and 8 times higher one-year mortality than those with a score of <2. We also found a strong correlation between each component of the qSOFA score and in-hospital mortality. This study found the qSOFA score to be a simple and useful tool for assessing patients with invasive listeriosis. Patients with scores of ≥2 should receive more aggressive treatment to reduce the high mortality rate, starting with antibiotics as soon as possible, such as ampicillin (with or without gentamicin), penicillin G, piperacillin–tazobactam, amoxicillin–clavulanate, meropenem, linezolid, and trimethoprim–sulfamethoxazole [11,12].

Leukocytosis is commonly seen in individuals with invasive listeriosis, but it does not differ between survivors and non-survivors. Patients with listeriosis frequently have increased CRP levels (up to 80%) [20], but it remains unclear if these levels can predict in-hospital death. Lobo et al. found that high CRP levels upon ICU admission are linked to a greater risk of organ failure and death, with persistently high CRP levels indicating poor outcomes in ICU patients [23]. However, Hung et al. showed that while 44.8% (13 of the 29) of listeriosis patients had CRP levels above 100 mg/L, it did not predict in-hospital mortality [24]. In the present study, higher CRP levels at admission and after 7 days were significantly associated with worse hospital outcomes. More research is needed to understand the relationship between CRP and in-hospital mortality from listeriosis. For now, we recommend monitoring CRP levels to identify patients who may need more aggressive treatment to prevent complications.

Researchers have linked hypoalbuminemia to 30-day all-cause mortality, the length of hospital admissions, and complications, suggesting that it may be used to identify patients at higher risk of poor outcomes [25,26]. However, the role of albumin in predicting death in listeriosis is poorly understood. In the current study, patients with invasive listeriosis commonly had hypoalbuminemia (2.86 ± 0.68 g/dL on average at admission and 2.62 ± 0.49 g/dL on day 7). The univariate analysis shows a strong link between more severe hypoalbuminemia on days 1 and 7 and in-hospital mortality. We are the first to show this connection in listeriosis, and we recommend using albumin levels to identify high-risk patients. The benefit of albumin replacement treatment for these individuals remains unclear and needs further investigation [27].

In patients with severe sepsis, elevated serum bilirubin levels have been linked to higher mortality. Patel et al. found that high bilirubin levels within 72 h of admission were associated with increased mortality in severe sepsis and septic shock patients [28]. Similarly, Yang et al. found that patients with bilirubin levels of ≥2 mg/dL had a 32.2% mortality rate compared with 24.8% for those with levels of <2 mg/dL [29]. In line with these studies, we found that higher bilirubin levels on day seven after admission were strongly associated with in-hospital death in listeriosis patients (1.44 odds ratio). Severe infection-induced liver dysfunction may explain this, and we recommend monitoring bilirubin levels in listeriosis patients to identify those at high risk.

No controlled trials have determined the best drug or treatment duration for *listeria* infections. Treatment recommendations are based on in vitro testing, animal studies, and clinical experience, with a small number of patients compared with historical controls. Generally, intravenous ampicillin is recommended for at least two weeks for bacteremia, while ampicillin plus gentamicin for at least three weeks is advised for meningitis [6,12]. In our study, we found that shorter treatment durations were substantially linked to higher in-hospital mortality, with the average ampicillin treatment duration being 2.0 ± 1.6 weeks. One possible reason is that more severely ill patients may not complete the full ampicillin course before death. Furthermore, we observed no significant difference between survivors and non-survivors when we incorporated all effective antibiotics in the analysis and assessed treatment durations. This suggests that analyzing ampicillin alongside other antibiotics may obscure its effect, underscoring the importance of making ampicillin the primary treatment for invasive listeriosis. We recommend using appropriate antibiotics, with a particular emphasis on ampicillin, and ensuring sufficient treatment durations to reduce mortality from invasive listeriosis.

Delays in therapy initiation have been linked to poor outcomes. A retrospective case-control study of 36 patients with *Listeria* meningitis showed a higher risk of death (hazard ratio 2.78; 95% CI 1.13–6.87) if antibiotic therapy was delayed more than six hours [30]. Another study of 100 CNS listeriosis cases found a link between delayed treatment and neurological problems and death (OR 1.07; 95% CI 1.01–1.16) [31]. Similarly, we found that longer delays in starting ampicillin therapy were significantly associated with higher in-hospital mortality. To reduce mortality in invasive listeriosis, we recommend starting appropriate antibiotics early and completing at least the minimum treatment duration.

Gentamicin is often used to treat listeriosis, particularly in meningitis cases, but its effectiveness remains uncertain owing to a lack of supporting randomized controlled trials. Some studies suggest that aminoglycosides may improve outcomes in listeriosis treatment. One study of 36 patients with invasive listeriosis found that those treated with gentamicin in combination with other therapy had a much lower 90-day mortality rate (10%) compared with those on monotherapy (60%), with a hazard ratio of 0.07 (95% CI: 0.01–0.53, *p* = 0.01) [11]. Another study of 818 cases across 372 centers showed that patients treated with aminoglycosides plus beta-lactams had higher survival rates (69% vs. 52%, *p* < 0.0001) [9]. The results of this study show that using amoxicillin and gentamicin together as a first-line therapy is supported by their ability to work together and kill bacteria in vitro, with a protective odds ratio of 0.35 (95% CI 0.22–0.56, *p* < 0.0001) for treatments lasting more than three days. These results show that using aminoglycosides along with beta-lactams might help treat invasive listeriosis. However, several studies hold the opposite view, suggesting that using aminoglycosides to treat *Listeria monocytogenes* infections may actually increase mortality. In a study of 46 patients with Listeria meningitis, those who received ampicillin plus gentamicin had a higher mortality rate (67%) than those treated with ampicillin alone (32%) (*p* = 0.024) [32]. Another study of 102 patients found that combining β-lactam and aminoglycosides increased the risk of early death (27.3%) compared with β-lactam therapy alone (4.3%) (*p* = 0.003) [33]. The nephrotoxicity of aminoglycosides and their limited ability to penetrate the central nervous system may explain these results, possibly worsening existing health conditions. Current guidelines no longer recommend using gentamicin and ampicillin together for invasive listeriosis [11]. Consistent with these findings, our study also found no difference in survival between patients treated with ampicillin alone and those who received the combination therapy. Therefore, we do not recommend the routine use of gentamicin combination therapy and suggest further trials to determine the best treatment approach.

In our study, most combined bloodstream infections were caused by CoNS (n = 9, 7.6%), followed by *E. coli* (n = 6, 5.1%) and *Candida* (n = 5, 4.2%). Bouza et al.’s analysis of 139 mixed bacterial and *Candida* bloodstream infections found that these infections had a higher mortality risk than polybacterial infections [34]. Conversely, our study found no difference in mortality between patients with or without combined *Listeria* and *Candida* bloodstream infections. Additionally, we also observed that patients with combined *E. coli* bacteremia had a higher mortality rate than those without. More research is needed to better understand the clinical significance of interactions between *Listeria*, polybacteria, and *Candida* given the small sample size of our study population.

This study has several limitations. Firstly, as a retrospective study conducted at a single hospital with mostly Asian patients, the results may not be generalizable. Second, our study’s focus on a single center limited the number of cases analyzed, complicating the determination of significant differences between certain features. Given the small sample size, we could not include all potential risk factors in our analysis. Instead, we focused on clinically relevant indicators that are most commonly used. Third, because our hospital does not routinely analyze the serotype of *Listeria monocytogenes*, we could not obtain serotype data. The most common serotypes are 4b, 1/2a, and 1/2b, with 4b being the most associated with listeriosis outbreaks, indicating higher virulence [16,35]. However, guidelines recommend against using serologic tests to diagnose listeriosis because of their poor sensitivity and specificity [36], so not testing for serotypes in our study aligns with these recommendations. Fourth, the government’s health policy may influence the effective treatment of listeriosis, as early identification and understanding are essential. Further research is needed to examine how these policies impact the prevalence and mortality of invasive listeriosis. Despite these limitations, this study contributes valuable knowledge regarding characteristics that may increase the risk of death among patients with invasive listeriosis.

## 5. Conclusions

Only 63.6% of patients with invasive listeriosis survived their initial hospitalizations. One year later, 39 (33.1%) of the hospitalized patients remained alive. In-hospital death rates were higher for people with peptic ulcer disease; confusion; shortness of breath; nausea or vomiting; muscle pain; headaches; shock; respiratory failure; mental changes; tachypnea (RR ≥ 22); a qSOFA score of 2 or more; higher BUN/CRP and lower albumin on the first day of hospitalization; higher PMN/ANC/BUN/potassium/CRP/total bilirubin and lower albumin after 7 days; a shorter ampicillin use duration; a longer treatment delay for ampicillin therapy; and combined *E. coli* bacteremia.

After multivariate analysis, respiratory failure, a qSOFA score of ≥2, and the length of the ampicillin prescription were all independently linked to worse in-hospital outcomes; a qSOFA score of ≥2 and the length of the ampicillin prescription were both separately linked to higher one-year mortality. A qSOFA score of ≥2 may identify high-risk individuals with invasive listeriosis, and we recommend using it as a preliminary mortality risk indicator. This study highlighted the characteristics of high-risk invasive listeriosis in patients and emphasized the significance of early identification and adequate antibiotic therapy.

## Figures and Tables

**Table 1:** Demographics of hospitalized patients with invasive listeriosis.

	Total (N = 118)	In-Hospital Survival (N = 75)	In-Hospital Death (N = 43)		
Variable	No. (%)			*p*-Value	Odds Ratio (95% CI)
**Age (years) (mean ± SD)**	60.0 ± 18.4	58.7 ± 18.5	62.4 ± 18.3	0.294	1.01 (0.99–1.03)
**Gender**					
Male	55 (46.6)	37 (49.3)	18 (41.9)	0.434	0.74 (0.34–1.57)
Female	63 (53.4)	38 (50.7)	25 (58.1)		
**Comorbidities**					
Diabetes mellitus	31 (26.3)	22 (29.3)	9 (20.9)	0.320	0.64 (0.25–1.51)
Hypertension	56 (47.5)	36 (48.0)	20 (46.5)	0.876	0.94 (0.44–2.00)
Coronary artery disease	7 (5.9)	4 (5.3)	3 (7.0)	0.717	1.33 (0.25–6.33)
Heart failure	19 (16.1)	11 (14.7)	8 (18.6)	0.576	1.33 (0.47–3.60)
Atrial fibrillation	15 (12.7)	10 (13.3)	5 (11.6)	0.789	0.86 (0.25–2.60)
Cirrhosis	23 (19.5)	11 (14.7)	12 (27.9)	0.085	2.25 (0.89–5.75)
Peptic ulcer disease	43 (36.4)	20 (26.7)	23 (53.5)	0.004 *	3.16 (1.45–7.05)
Cerebral vascular accident	11 (9.3)	7 (9.3)	4 (9.3)	0.996	1.00 (0.25–3.52)
Chronic obstructive airway disease	10 (8.5)	6 (8.0)	4 (9.3)	0.807	1.18 (0.29–4.38)
Solid cancer	39 (33.1)	24 (32.0)	15 (34.9)	0.749	1.14 (0.51–2.51)
Hematologic malignancy	20 (16.9)	16 (21.3)	4 (9.3)	0.103	0.38 (0.10–1.12)
Autoimmune diseases	29 (24.6)	18 (24.0)	11 (25.6)	0.848	1.09 (0.45–2.57)
Organ transplant	6 (5.1)	4 (5.3)	2 (4.7)	0.871	0.87 (0.12–4.64)
Chronic dialysis	26 (22.0)	14 (18.7)	12 (2, 9)	0.246	1.69 (0.69–4.10)
**Immunosuppressants use**	67 (56.8)	42 (56.0)	25 (58.1)	0.821	1.09 (0.51–2.35)
**Anti-cancer therapy**					
Chemotherapy in prior 4 weeks	15 (12.7)	7 (9.3)	8 (18.6)	0.153	2.22 (0.74–6.82)
Radiotherapy in prior 4 weeks	12 (10.2)	7 (9.3)	5 (11.6)	0.692	1.28 (0.36–4.28)

**Notes:** * *p*-value < 0.05. The *p*-value represents a comparison of risk factors between in-hospital survival and death groups. The odds ratio (OR) compares the odds of mortality in the in-hospital death group to the odds in the survival group. **Abbreviations:** CI, confidence interval; SD, standard deviation.

**Table 2:** Clinical characteristics of hospitalized patients with invasive listeriosis.

	Total (N = 118)	In-Hospital Survival (N = 75)	In-Hospital Death (N = 43)		
Characteristics	No. (%)			*p*-Value	Odds Ratio (95% CI)
**Symptoms and signs**					
Fever	111 (94.1)	69 (92.0)	42 (97.7)	0.238	3.65 (0.60–70.24)
Nausea or vomiting	80 (67.8)	57 (76.0)	23 (53.5)	0.013 *	0.36 (0.16–0.80)
Myalgia	52 (44.1)	40 (53.3)	12 (27.9)	0.008 *	0.34 (0.15–0.74)
Abdominal pain	46 (39.0)	27 (36.0)	19 (44.2)	0.381	1.41 (0.65–3.03)
Diarrhea	39 (33.1)	28 (37.3)	11 (25.6)	0.194	0.58 (0.24–1.30)
Headache	37 (31.4)	31 (41.3)	6 (14.0)	0.003 *	0.23 (0.08–0.58)
Confusion	53 (44.9)	14 (18.7)	39 (90.7)	0.000 *	42.48 (14.38–160.00)
Dyspnea	66 (55.9)	35 (46.7)	31 (72.1)	0.008 *	2.95 (1.34–6.80)
**Complications**					
Shock	38 (32.2)	9 (12)	29 (67.4)	0.000 *	15.19 (6.14–41.08)
Respiratory failure	50 (42.4)	13 (17.3)	37 (86.1)	0.000 *	29.41 (10.97–91.50)
SBP ≤ 100 (mmHg)	51 (43.2)	16 (21.3)	35 (81.4)	0.000 *	16.13 (6.55–44.05)
Altered mental status	53 (44.9)	14 (18.7)	39 (90.7)	0.000 *	42.48 (14.38–160.00)
RR ≥ 22 (BPM)	56 (47.5)	16 (21.3)	40 (93.0)	0.000 *	49.17 (15.39–222.52)
qSOFA score ≥ 2	45 (38.1)	3 (7.0)	70 (93.3)	0.000 *	186.67 (48.96–1005.23)
**Laboratory data (mean ± SD)**					
*Hospital Day 1*					
WBC (k/μL)	11.92 ± 13.93	12.67 ± 16.83	10.63 ± 6.22	0.455	0.99 (0.95–1.02)
Hemoglobin (g/dL)	10.54 ± 2.69	10.71 ± 2.78	10.23 ± 2.52	0.352	0.93 (0.81–1.08)
Platelet (k/μL)	157.34 ± 92.07	160.88 ± 90.11	151.16 ± 96.16	0.580	1.00 (0.99–1.00)
PMN (%)	76.88 ± 18.70	75.20 ± 20.01	79.81 ± 15.97	0.207	1.02 (0.99–1.04)
ANC (/μL)	7979.34 ± 5640.54	7540.71 ± 5474.45	8744.40 ± 5906.19	0.267	1.00 (1.00–1.00)
Lymphocyte (%)	12.22 ± 10.44	12.51 ± 9.33	11.72 ± 12.23	0.693	0.99 (0.95–1.03)
BUN (mg/dL)	43.50 ± 45.14	35.84 ± 36.39	56.71 ± 55.25	0.032 *	1.01 (1.00–1.02)
Creatinine (mg/dL)	2.76 ± 3.16	2.45 ± 2.90	3.30 ± 3.53	0.171	1.09 (0.96–1.23)
Sodium (mEq/L)	134.36 ± 6.12	134.74 ± 5.95	133.69 ± 6.42	0.376	0.97 (0.91–1.03)
Potassium (mEq/L)	4.03 ± 0.78	4.00 ± 0.82	4.09 ± 0.72	0.552	1.16 (0.71–1.90)
CRP (mg/L)	91.76 ± 83.96	71.19 ± 70.60	125.56 ± 93.61	0.002 *	1.01 (1.00–1.01)
Albumin (g/dL)	2.86 ± 0.68	3.05 ± 0.67	2.59 ± 0.60	0.004 *	0.31 (0.13–0.66)
Total bilirubin (mg/dL)	1.45 ± 2.10	1.12 ± 1.23	1.88 ± 2.83	0.104	1.21 (0.98–1.58)
HbA1c (%)	6.65 ± 1.29	6.87 ± 1.40	5.93 ± 0.40	0.283	0.47 (0.07–1.50)
*Hospital Day 7*					
WBC (k/μL)	10.07 ± 9.55	9.54 ± 10.88	11.02 ± 6.58	0.448	1.02 (0.97–1.07)
Hemoglobin (g/dL)	10.13 ± 2.07	10.16 ± 2.05	10.08 ± 2.13	0.831	0.98 (0.81–1.18)
Platelet (k/μL)	159.35 ± 94.81	173.94 ± 72.26	142.94 ± 113.62	0.099	
PMN (%)	75.59 ± 19.61	71.64 ± 21.41	82.60 ± 13.55	0.008 *	1.04 (1.01–1.08)
ANC (/μL)	7162.42 ± 5052.81	5820.09 ± 3649.37	9511.50 ± 6236.94	0.001 *	1.00 (1.00–1.00)
Lymphocyte (%)	14.44 ± 12.73	15.98 ± 11.35	11.70 ± 14.63	0.096	0.97 (0.93–1.00)
BUN (mg/dL)	38.55 ± 35.73	29.41 ± 24.17	53.62 ± 45.73	0.004 *	1.01 (1.01–1.04)
Creatinine (mg/dL)	2.31 ± 2.95	1.96 ± 2.60	2.93 ± 3.44	0.115	1.12 (0.98–1.29)
Sodium (mEq/L)	138.34 ± 5.79	138.43 ± 4.58	138.2 ± 7.41	0.835	0.99 (0.93–1.06)
Potassium (mEq/L)	3.94 ± 0.73	3.80 ± 0.59	4.15 ± 0.87	0.021 *	2.00 (1.14–3.76)
CRP (mg/L)	70.53 ± 74.97	56.38 ± 64.23	94.62 ± 85.95	0.020 *	1.01 (1.00–1.01)
Albumin (g/dL)	2.62 ± 0.49	2.84 ± 0.50	2.38 ± 0.35	0.002 *	0.07 (0.01–0.31)
Total bilirubin (mg/dL)	1.84 ± 3.38	0.93 ± 0.80	3.11 ± 4.93	0.033 *	1.44 (1.11–2.18)
**Treatment**					
Ampicillin duration (weeks)	2.0 ± 1.6	2.2 ± 1.6	1.6 ± 1.5	0.044 *	0.76 (0.58–0.98)
All effective antibiotics duration (weeks)	2.6 ± 1.4	2.6 ± 1.4	2.5 ± 1.4	0.813	1.00 (0.96–1.03)
Gentamicin synergy	26 (22.0)	20 (26.7)	6 (14.0)	0.155	0.45 (0.15–1.16)
Treatment delay (days) ^Δ^	1.4 ± 2.2	1.0 ± 1.9	2.1 ± 2.5	0.015 *	1.26 (1.06–1.54)
**Blood culture results**					
Listeria monocytogenes	115 (97.5)	73 (97.3)	42 (97.7)	0.910	1.15 (0.11–25.20)
Escherichia coli	6 (5.1)	1 (1.3)	5 (11.6)	0.041 *	9.74 (1.50–190.12)
Klebsiella pneumonia	2 (1.7)	2 (2.67)	0 (0)	0.988	N/A
Enterobacter cloacae	1 (0.8)	0 (0)	1 (2.3)	0.991	N/A
Pseudomonas aeruginosa	1 (0.8)	0 (0)	1 (2.3)	0.991	N/A
MRSA	1 (0.8)	0 (0)	1 (2.3)	0.988	N/A
CoNS	9 (7.6)	3 (4.0)	6 (14.0)	0.065	3.89 (0.97–19.27)
Candida	5 (4.2)	2 (2.7)	3 (7.0)	0.281	2.74 (0.44–21.44)
**CSF culture results**					
Listeria monocytogenes	11 (9.3)	7 (9.3)	4 (9.3)	0.996	1.00 (0.25–3.52)

**Notes:** * *p*-value < 0.05; ^Δ^ interval between the confirmation of diagnosis and the initiation of ampicillin. The *p*-value represents a comparison of risk factors between in-hospital survival and death groups. The odds ratio (OR) compares the odds of mortality in the in-hospital death group to the odds in the survival group. **Abbreviations:** CI, confidence interval; N/A, not applicable; SBP, systolic blood pressure; RR, respiratory rate; BPM, beats per minute; qSOFA, quick Sequential Organ Failure Assessment; SD, standard deviation; WBC, white blood count; PMN, polymorphonuclear leukocytes; ANC, absolute neutrophil count; CRP, C-reactive protein; MRSA, Methicillin-resistant Staphylococcus aureus; CoNS, Coagulase-negative Staphylococcus.

**Table 3:** Multivariate analysis of factors for in-hospital and one-year mortality in hospitalized patients with invasive listeriosis.

Population	Variable	Risk Measurement	*p*-Value
In-Hospital Mortality		Odds Ratio (95% CI)	
	qSOFA score ≥ 2	106.59 (17.26–1136.26)	0.000 *
	Shock	1.48 (0.13–13.82)	0.733
	Respiratory failure	7.58 (1.14–52.69)	0.031 *
	Duration of ampicillin	0.53 (0.31–0.84)	0.012 *
	Blood culture *E. coli*	0.35 (0.02–10.73)	0.478
	Treatment delay	1.18 (0.82–1.69)	0.345
**1-year mortality**		**Hazard ratio (95% CI)**	
	qSOFA score ≥ 2	8.46 (3.84–18.64)	0.000 *
	Shock	1.89 (0.99–3.63)	0.054
	Respiratory failure	0.93 (0.44–1.97)	0.849
	Duration of ampicillin	0.65 (0.54–0.78)	0.000 *
	Blood culture *E. coli*	1.26 (0.48–3.30)	0.639
	Treatment delay	0.96 (0.85–1.09)	0.544

**Notes:** * *p*-value < 0.05. **Abbreviations:** CI, confidence interval; qSOFA, quick Sequential Organ Failure Assessment. Logistic regression for in-hospital mortality Cox regression for 1-year mortality.

## Data Availability

The original data presented in the study are openly available in OSFHOME: https://osf.io/yhvzp/?view_only=9039734851c04cc48d3c0f604616beb1, accessed on 23 September 2024.
